# Surgical management and limb salvage outcomes in orthopedic trauma patients with concomitant popliteal artery injury: a single-center retrospective analysis

**DOI:** 10.3389/fsurg.2026.1961253

**Published:** 2026-09-22

**Authors:** Huagang Zhang, Shengqiang Fei, Yingcheng Bai, Chunhong Xu, Wei Li, Hongchun Sun, Fengduo Zhang

**Affiliations:** 1 Department of Burn and Plastic Surgery, No.971 Hospital of the People’s Liberation Army Navy, Qingdao, China; 2 Department of Hand Surgery Anesthesia, No.971 Hospital of the People’s Liberation Army Navy, Qingdao, China; 3 Department of General Surgery, No.971 Hospital of the People’s Liberation Army Navy, Qingdao, China; 4 Department of Endocrinology, No.971 Hospital of the People’s Liberation Army Navy, Qingdao, China; 5 Department of Hand Surgery Rehabilitation, No.971 Hospital of the People’s Liberation Army Navy, Qingdao, China; 6 Department of Emergency Medicine, No.971 Hospital of the People’s Liberation Army Navy, Qingdao, China

**Keywords:** amputation, fasciotomy, fracture fixation, limb salvage, multidisciplinary care, orthopedic trauma, popliteal artery injury, vascular reconstruction

## Abstract

**Background:**

Traumatic popliteal artery injury often occurs with complex orthopedic and soft-tissue damage and requires coordinated vascular, skeletal, decompressive, and wound management. We evaluated real-world treatment patterns and limb-salvage outcomes in a single-center cohort of patients with combined orthopedic trauma and popliteal artery injury.

**Methods:**

We retrospectively reviewed 48 hospitalization records from 46 patients treated between 2002 and 2025. Repeated hospitalizations related to the same injury were reconciled into 46 independent patient/injury episodes. The primary outcome was limb salvage, defined as avoidance of amputation during the complete injury episode. Primary amputations were described separately, and treatment-associated analyses were restricted to the 42 episodes in which limb salvage was attempted. Vascular, orthopedic, and adjunct procedures were summarized descriptively; selected procedure-associated secondary-amputation rates were compared using Fisher's exact test. Laboratory analyses used variable-specific available cases without imputation.

**Results:**

Nine of 46 injury episodes ended in amputation: four were primary amputations and five occurred after attempted salvage. Among the 42 salvage-attempted episodes, 37 limbs were preserved, yielding an observed limb-salvage rate of 88.1%. Formal vascular reconstruction was performed in 27 episodes, including 17 vein graft reconstructions and 10 direct arterial repairs. Limb salvage was observed in 15 of 17 vein graft cases (88.2%) and 9 of 10 direct repairs (90.0%); these rates were considered descriptive rather than comparative. No selected orthopedic or adjunct procedure was significantly associated with secondary amputation. Procedures frequently occurred in combination, particularly vascular exploration with debridement or external fixation, highlighting the integrated nature of limb-salvage care. Admission laboratory comparisons were limited by substantial missingness and showed no significant between-group differences.

**Conclusions:**

In this retrospective cohort of traumatic popliteal artery injury, limb salvage was achieved in most patients for whom salvage was attempted. Outcomes after vein graft and direct repair were favorable descriptively, but the small, clinically selected groups do not support comparative efficacy conclusions. Vein graft and direct repair each achieved favorable, though non-comparable, salvage rates, and the frequent overlap of vascular, orthopedic, decompressive, and soft-tissue procedures indicates that limb salvage in this setting is delivered through coordinated, multidisciplinary care rather than any single intervention. These procedure-specific observations remain exploratory and warrant confirmation in larger cohorts with standardized measures of injury severity and ischemia time.

## Introduction

Trauma patients with concomitant arterial injury represent a distinct subgroup within orthopedic trauma because limb outcome depends not only on fracture stabilization but also on timely perfusion restoration, management of reperfusion-related swelling, and durable soft-tissue coverage. Despite advances in vascular technique and damage-control principles, compartment syndrome and infection, along with complex wound failure, continue to drive limb loss or unplanned reoperation. Clinical decision-making grows especially difficult when limb viability is equivocal, ischemia time is prolonged, or multisystem injury necessitates staged procedures.

The contemporary literature identifies several recurring determinants of outcome. Ischemia duration, combined venous and nerve injury, and extent of soft-tissue destruction are among the strongest predictors of limb loss, with popliteal artery injuries carrying the highest risk (1–4). Temporary intravascular shunting has emerged as a bridge to definitive repair in damage-control settings, shortening warm ischemia while permitting resuscitation and skeletal stabilization (5–8). Compartment syndrome and the timing of fasciotomy remain central concerns: delayed decompression may escalate tissue necrosis and secondary amputation risk, yet the threshold for prophylactic fasciotomy varies with mechanism, ischemia time, and the extent of combined injury (9–12). Orthopedic stabilization strategies and wound adjuncts, including negative pressure wound therapy (NPWT), serial debridement, and grafting, further shape limb salvage trajectories in multidisciplinary settings (13–16).

Most published series, however, are restricted to isolated vascular injury cohorts or selected operative populations and seldom examine how vascular repair, orthopedic fixation, and soft-tissue management converge within limb-salvage pathways. This limitation matters because treatment decisions in combined injuries are inherently interdependent: a single injury episode may encompass several overlapping procedures whose individual contributions to outcome are difficult to isolate.

We therefore undertook a retrospective exploratory study to characterize clinical profiles, multidisciplinary treatment pathways, and limb-salvage outcomes in patients with orthopedic trauma and concomitant popliteal artery injury. We additionally examined procedure co-occurrence and temporal patterns to describe evolving practice within a single institution.

## Methods

### Study design and setting

This retrospective observational study included patients with orthopedic trauma and concomitant arterial injury who were treated at a single tertiary care center between 2002 and 2025. The study was conducted and reported in accordance with the STROBE recommendations for observational studies. The Ethics Committee of No. 971 Hospital of the People's Liberation Army Navy approved the study and waived the requirement for informed consent because of its retrospective design (No. 971LL2025031707).

## Study population and construction of injury episodes

Hospitalization records were eligible if they involved (1) orthopedic trauma requiring inpatient treatment and (2) a documented concomitant arterial injury that underwent vascular evaluation or intervention. The source dataset contained 48 hospitalization records from 46 patients.

Because two patients had more than one hospitalization related to the same underlying limb injury, hospitalization records were reconciled at the patient/injury level before analysis. In one patient, a same-day discharge and readmission separated by only several minutes represented an administrative continuation of the same hospitalization rather than a clinical readmission. In the other, a subsequent hospitalization was related to the original injury and involved additional vascular and orthopedic intervention. Records belonging to the same injury were therefore linked into a single episode, with the first hospitalization designated as the index admission. The final analytic cohort comprised 46 independent patient/injury episodes represented by 48 hospitalization records.

Review of the diagnosis and operative records confirmed that all included episodes involved the lower extremity and the popliteal artery. Age, sex, and treatment era were defined from the index admission. For episodes spanning more than one hospitalization, inpatient days and hospital costs were summed across the linked records.

## Outcome definitions

The primary clinical outcome was limb salvage, defined as avoidance of amputation during the complete injury episode. Amputations were further classified as primary or secondary. Primary amputation was defined as amputation without a documented preceding limb-salvage attempt during the injury episode. Secondary amputation referred to amputation after a documented or clinically adjudicated attempt at limb salvage or prior vascular treatment.

Analyses of treatment patterns and observed treatment-associated outcomes were restricted to the salvage-attempted cohort, defined as all injury episodes other than primary amputation. Primary amputations were described separately and were not treated as a vascular management strategy for comparative analyses.

Injury-related readmission requiring additional operative intervention was recorded separately as a secondary descriptive outcome and was not incorporated into the limb-salvage endpoint. Administrative continuation of an index hospitalization was not classified as readmission.

## Data collection and clinical variables

Clinical variables were derived from structured administrative, laboratory, and imaging records together with free-text operative documentation. Operative descriptions were initially screened using predefined text-based rules and subsequently reviewed at the episode level to resolve ambiguous vascular procedures and amputation status. Where automated classification conflicted with the operative record, the clinically adjudicated classification was retained.

Demographic and utilization variables included age, sex, injury side, treatment era, cumulative inpatient days, and cumulative in-hospital costs. Treatment era was defined according to the year of index admission as 2002–2012 or 2013–2025.

## Vascular management

Vascular procedures were characterized at the injury-episode level. Formal vascular reconstruction was defined as either autologous vein graft reconstruction or direct arterial repair. Vein graft and direct repair were retained as separate subgroups.

Episodes without formal reconstruction were not combined into a single treatment category because they represented clinically distinct circumstances. These pathways included vascular exploration without reconstruction, wound-management-only episodes, absence of a documented vascular procedure, other or uncertain vascular repair/procedures, and prior vascular treatment for which the specific technique could not be reliably determined. Primary amputation was recorded separately.

Observed limb-salvage rates after vein graft and direct repair were summarized descriptively. No equivalence or comparative efficacy analysis between reconstruction techniques was performed.

## Orthopedic and adjunct procedures

Fracture fixation was considered present when internal fixation, external fixation, or both were performed during the injury episode. Cast or splint treatment alone was not classified as fracture fixation. Adjunct procedures were coded separately and included fasciotomy, negative-pressure wound therapy (NPWT), skin grafting, debridement, and nerve repair. For patients with more than one linked hospitalization, a procedure was considered present if it was performed during any hospitalization within the same injury episode.

To describe the overall procedural burden without implying a validated severity scale, an exploratory unweighted procedure-burden count was calculated from seven predefined domains: vascular reconstruction or repair, vascular exploration, internal fixation, external fixation, fasciotomy, NPWT, and skin grafting or nerve repair. The resulting count ranged from 0 to 7 and was used only as a descriptive exploratory measure.

## Admission laboratory parameters

Laboratory analyses used the first available value obtained within 48 h of the index admission. The prespecified laboratory variables were white blood cell count (WBC), hemoglobin, platelet count, prothrombin time (PT), serum albumin, and the neutrophil-to-lymphocyte ratio (NLR). Values obtained outside the 48-hour index-admission window were not substituted when an early value was unavailable.

Laboratory missingness was reported for each variable. Analyses used a variable-specific available-case approach, and missing values were not imputed.

## Statistical analysis

Continuous variables are reported as median and interquartile range (IQR), and categorical variables as number and percentage. In the full cohort, demographic, utilization, and laboratory variables were compared between episodes ending in limb salvage and those ending in amputation using the Wilcoxon rank-sum test for continuous variables and Fisher's exact test for categorical variables.

Treatment-associated analyses were conducted primarily in the 42 salvage-attempted episodes. For selected orthopedic and adjunct procedures, the observed secondary-amputation rate was calculated among episodes with and without the procedure and compared using Fisher's exact test. These comparisons were unadjusted and exploratory and were not interpreted as estimates of causal treatment effects. The exploratory procedure-burden count was compared with the Wilcoxon rank-sum test.

Outcomes after vein graft and direct arterial repair were reported descriptively as counts and percentages without formal testing for equivalence or comparative efficacy. Procedure co-occurrence within the salvage-attempted cohort was summarized using pairwise counts to characterize commonly used multidisciplinary treatment combinations.

Temporal analyses compared an early era (2002–2012) with a recent era (2013–2025). Rates of amputation, primary amputation, formal vascular reconstruction, fracture fixation, and fasciotomy were compared in the full cohort, while secondary amputation was examined separately within the salvage-attempted cohort. Categorical era comparisons used Fisher's exact test.

Because only nine amputations occurred in the full cohort and five secondary amputations occurred among the 42 salvage-attempted episodes, multivariable regression was not performed. Time-to-event analyses were also not undertaken because reliable longitudinal time-to-event follow-up was not available across the full study period. Laboratory missing values were not imputed. All tests were two-sided. Given the small sample and exploratory design, *P* values were interpreted descriptively without adjustment for multiple comparisons, and treatment-related findings were considered hypothesis-generating rather than confirmatory. Analyses were performed using R version 4.6.

## Results

### Cohort characteristics and limb outcomes

The 48 hospitalization records represented 46 independent patient/injury episodes. All injuries involved the lower extremity and the popliteal artery. The median age at index admission was 42.0 years (IQR, 30.5–53.0), and 42 patients (91.3%) were male. Twenty-seven injuries (58.7%) involved the left limb, 16 (34.8%) the right limb, and three (6.5%) had unspecified laterality. Median cumulative inpatient stay was 24.0 days (IQR, 13.2–59.0), and median cumulative in-hospital cost was 64,238 CNY (IQR, 23,389–108,867) (Table 1; Figure 1).

**Figure 1:**
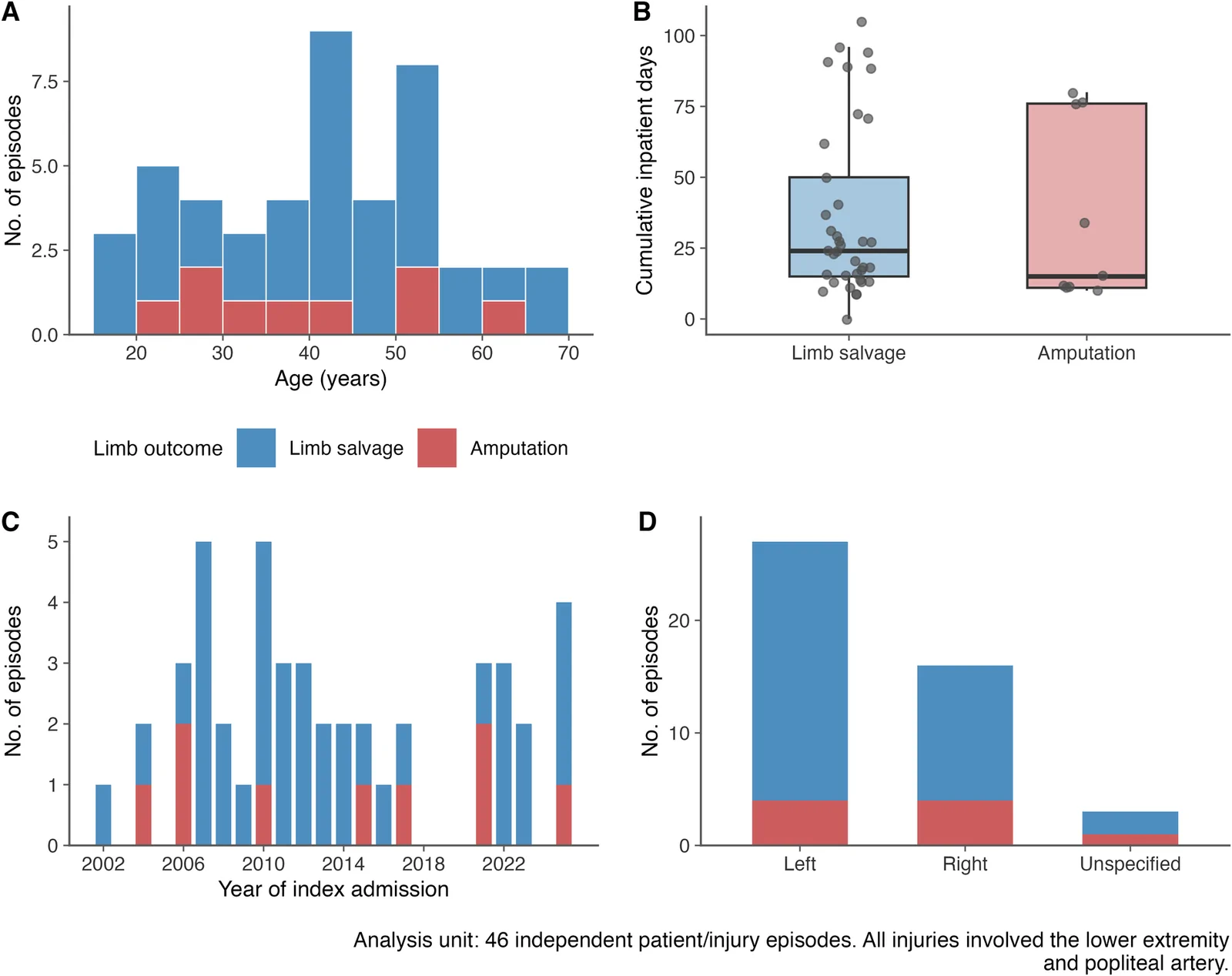
Cohort characteristics and temporal distribution. Characteristics of the 46 independent patient/injury episodes, stratified by final limb outcome. **(A)** Age distribution. **(B)** Cumulative inpatient days across all hospitalizations belonging to the same injury episode. **(C)** Distribution of index admissions across the study period. **(D)** Injury laterality. All included injuries involved the lower extremity and popliteal artery.

**Table 1:** Baseline characteristics of independent injury episodes according to limb outcome.

Variable	Limb salvage (*n* = 37)	Amputation (*n* = 9)	*P* value
Demographics			
Age, years, median (IQR)	43.0 (32.0–54.0)	40.0 (26.0–53.0)	0.678
Male sex, *n* (%)	35 (94.6)	7 (77.8)	0.167
Injury side, *n* (%)			0.400
Left	23 (62.2)	4 (44.4)	
Right	12 (32.4)	4 (44.4)	
Unspecified	2 (5.4)	1 (11.1)	
Treatment era and resource use			
Treatment era, *n* (%)			0.711
2002–2012	21 (56.8)	4 (44.4)	
2013–2025	16 (43.2)	5 (55.6)	
Cumulative inpatient days, median (IQR)	24.0 (15.0–50.0)	15.0 (11.0–76.0)	0.561
Cumulative in-hospital costs, CNY, median (IQR)	65,417 (23,559–109,577)	49,582 (23,183–78,448)	0.580

Data are presented at the level of 46 independent patient/injury episodes reconstructed from 48 hospitalization records. All injuries involved the lower extremity and popliteal artery. Continuous variables were compared using the Wilcoxon rank-sum test and categorical variables using Fisher's exact test. IQR, interquartile range.

Nine of the 46 injury episodes (19.6%) ended in amputation. Four were classified as primary amputations (8.7% of the full cohort), whereas five occurred after an attempted limb-salvage pathway. Limb salvage was therefore achieved in 37 of 46 episodes (80.4%) overall. After exclusion of the four primary amputations, 42 episodes constituted the salvage-attempted cohort; 37 of these limbs were salvaged, corresponding to an observed salvage rate of 88.1%.

One injury-related readmission requiring further vascular and orthopedic intervention was identified and was reported separately from the limb-salvage outcome. A second same-day hospitalization record represented administrative continuation of the index admission and was not classified as a clinical readmission.

Age, sex, injury side, treatment era, cumulative inpatient stay, and cumulative in-hospital costs did not differ significantly between episodes ending in limb salvage and those ending in amputation (all *P* > 0.05; Table 1).

## Treatment patterns among salvage-attempted episodes

Among the 42 salvage-attempted episodes, formal vascular reconstruction was performed in 27 (64.3%), comprising 17 vein graft reconstructions and 10 direct arterial repairs. Six episodes underwent vascular exploration without documented reconstruction, three involved wound management without a formal vascular reconstruction, and two had no documented vascular procedure. The remaining four episodes had other or incompletely specified vascular treatment pathways, including a recorded past vascular treatment event, the specific technique cannot be determined (Table 2; Figure 2A).

**Figure 2:**
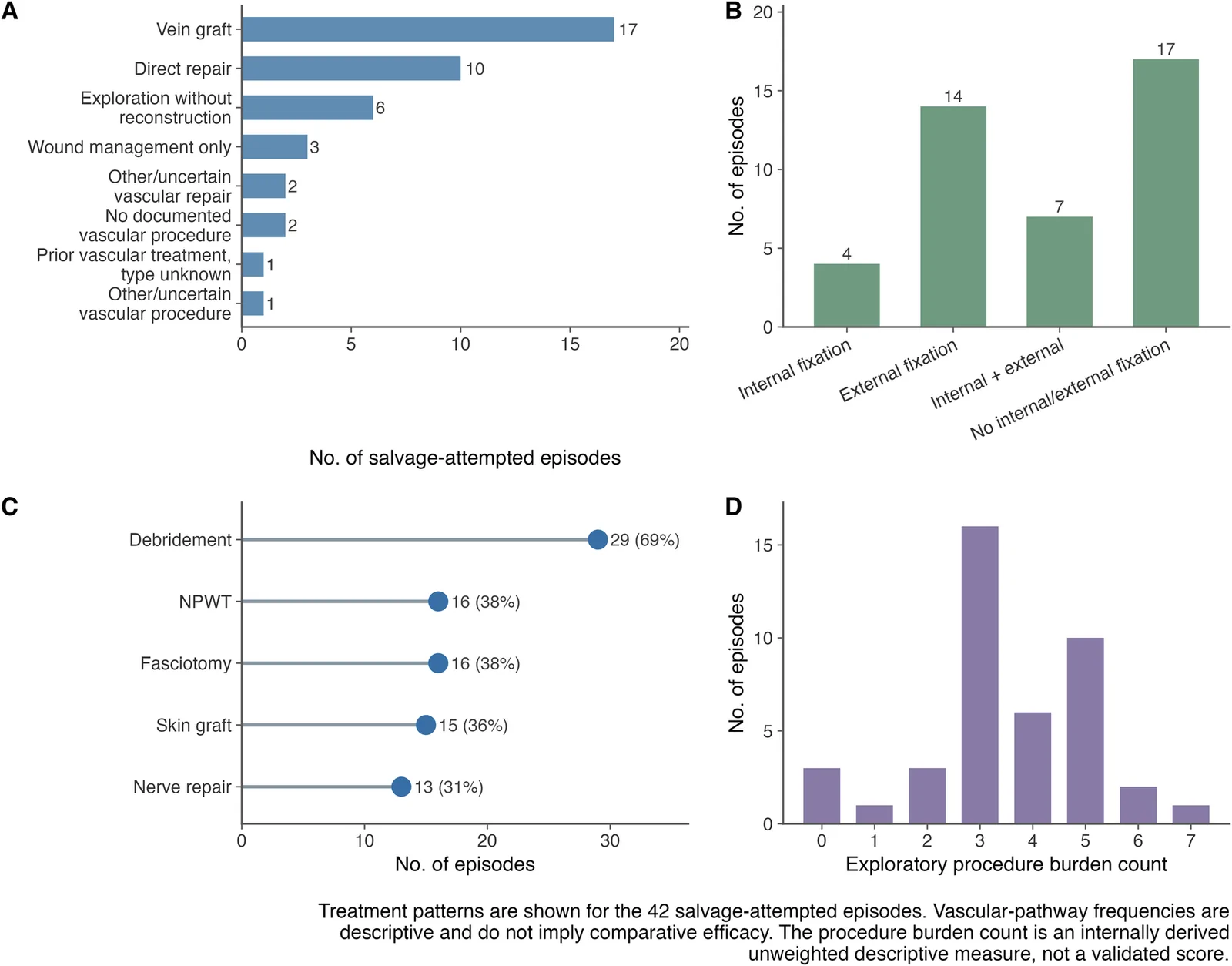
Treatment patterns among salvage-attempted injury episodes. Operative management among the 42 injury episodes in which limb salvage was attempted. **(A)** Documented vascular treatment pathways. Categories without formal reconstruction are shown separately because they represent heterogeneous clinical circumstances. **(B)** Fracture fixation patterns. **(C)** Frequency of selected adjunct procedures. **(D)** Distribution of the exploratory procedure-burden count. The procedure-burden count is an internally derived, unweighted descriptive measure and is not a validated severity or complexity score. NPWT, negative-pressure wound therapy.

**Table 2:** Vascular treatment pathways and observed limb outcomes among salvage-attempted injury episodes.

Vascular pathway	*n*	Limb salvage, *n* (%)	Secondary amputation, *n* (%)
Vein graft	17	15 (88.2)	2 (11.8)
Direct arterial repair	10	9 (90.0)	1 (10.0)
Exploration without reconstruction	6	6 (100.0)	0
Wound management only	3	3 (100.0)	0
No documented vascular procedure	2	2 (100.0)	0
Other/uncertain vascular repair	2	1 (50.0)	1 (50.0)
Other/uncertain vascular procedure	1	1 (100.0)	0
Prior vascular treatment, type unknown	1	0	1 (100.0)
Total	42	37 (88.1)	5 (11.9)

The table summarizes vascular treatment pathways among the 42 injury episodes in which limb salvage was attempted. Categories without formal reconstruction reflect heterogeneous clinical circumstances rather than a single treatment approach and are not directly comparable with one another or with vein graft and direct repair. No formal efficacy comparison was performed across pathways.

Internal fixation alone was used in four episodes, external fixation alone in 14, and combined internal and external fixation in seven; thus, 25 of 42 salvage-attempted episodes (59.5%) underwent internal and/or external fracture fixation (Figure 2B). Debridement was the most frequent adjunct procedure (29/42, 69.0%), followed by fasciotomy (16/42, 38.1%), NPWT (16/42, 38.1%), skin grafting (15/42, 35.7%), and nerve repair (13/42, 31.0%) (Figure 2C). The exploratory procedure-burden count had a median of 3 (IQR, 3–5) procedures per episode (Figure 2D).

## Observed secondary-amputation rates in the salvage-attempted cohort

Five secondary amputations occurred among the 42 episodes in which limb salvage was attempted. None of the selected treatment procedures showed a statistically significant difference in secondary-amputation rate on unadjusted analysis (Figure 3).

**Figure 3:**
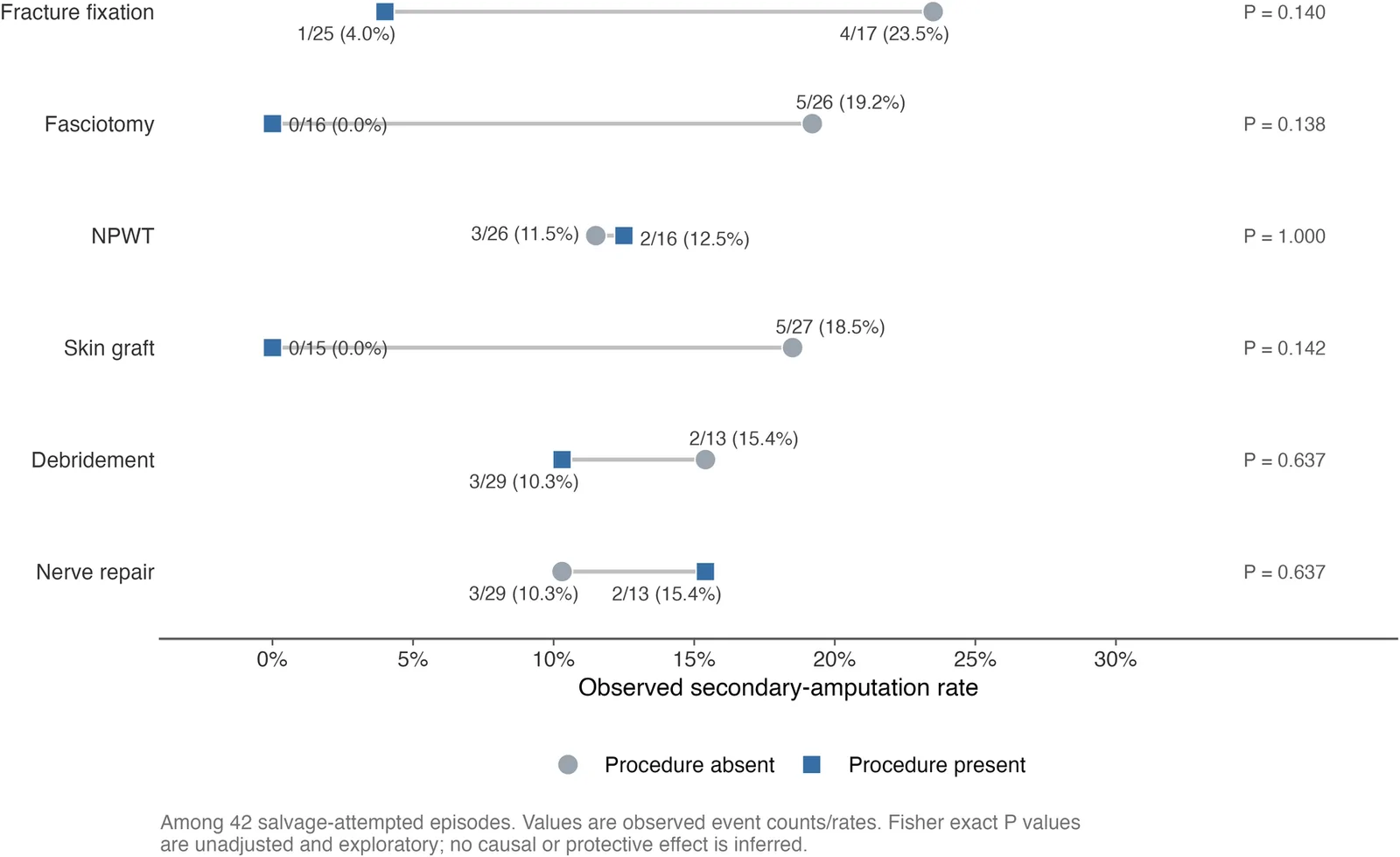
Observed secondary-amputation rates according to selected procedures. Secondary-amputation rates among the 42 salvage-attempted injury episodes according to the presence or absence of fracture fixation, fasciotomy, negative-pressure wound therapy (NPWT), skin grafting, debridement, and nerve repair. Labels show the number of secondary amputations, denominator, and observed percentage. *P* values were calculated using Fisher's exact test. Comparisons are unadjusted and exploratory and should not be interpreted as estimates of causal or protective treatment effects.

Secondary amputation occurred in 1 of 25 episodes with fracture fixation (4.0%) compared with 4 of 17 without fixation (23.5%; *P* = 0.140). No secondary amputations occurred among the 16 episodes in which fasciotomy was performed, compared with 5 of 26 without fasciotomy (19.2%; *P* = 0.138). Similarly, no secondary amputations occurred among the 15 episodes involving skin grafting, compared with 5 of 27 without skin grafting (18.5%; *P* = 0.142). These differences did not reach statistical significance. The observed rates were similar for NPWT (12.5% vs. 11.5%; *P* = 1.000), debridement (10.3% vs. 15.4%; *P* = 0.637), and nerve repair (15.4% vs. 10.3%; *P* = 0.637). These comparisons were exploratory and unadjusted. The underlying event counts and rates are shown in Figure 3.

The median exploratory procedure-burden count was 3 (IQR, 3–5) among salvaged limbs and 3 (IQR, 2–3) among episodes ending in secondary amputation (*P* = 0.111).

## Procedure co-occurrence and integrated treatment pathways

Procedures frequently occurred in combination rather than as isolated interventions (Figure 4). Vascular exploration co-occurred with debridement in 23 episodes and with external fixation in 17. Vein graft reconstruction was accompanied by vascular exploration in 15 episodes and by external fixation in 13. Other common combinations included external fixation with debridement (*n* = 15), fasciotomy with debridement (*n* = 14), and debridement with either NPWT or skin grafting (*n* = 13 for each combination).

**Figure 4:**
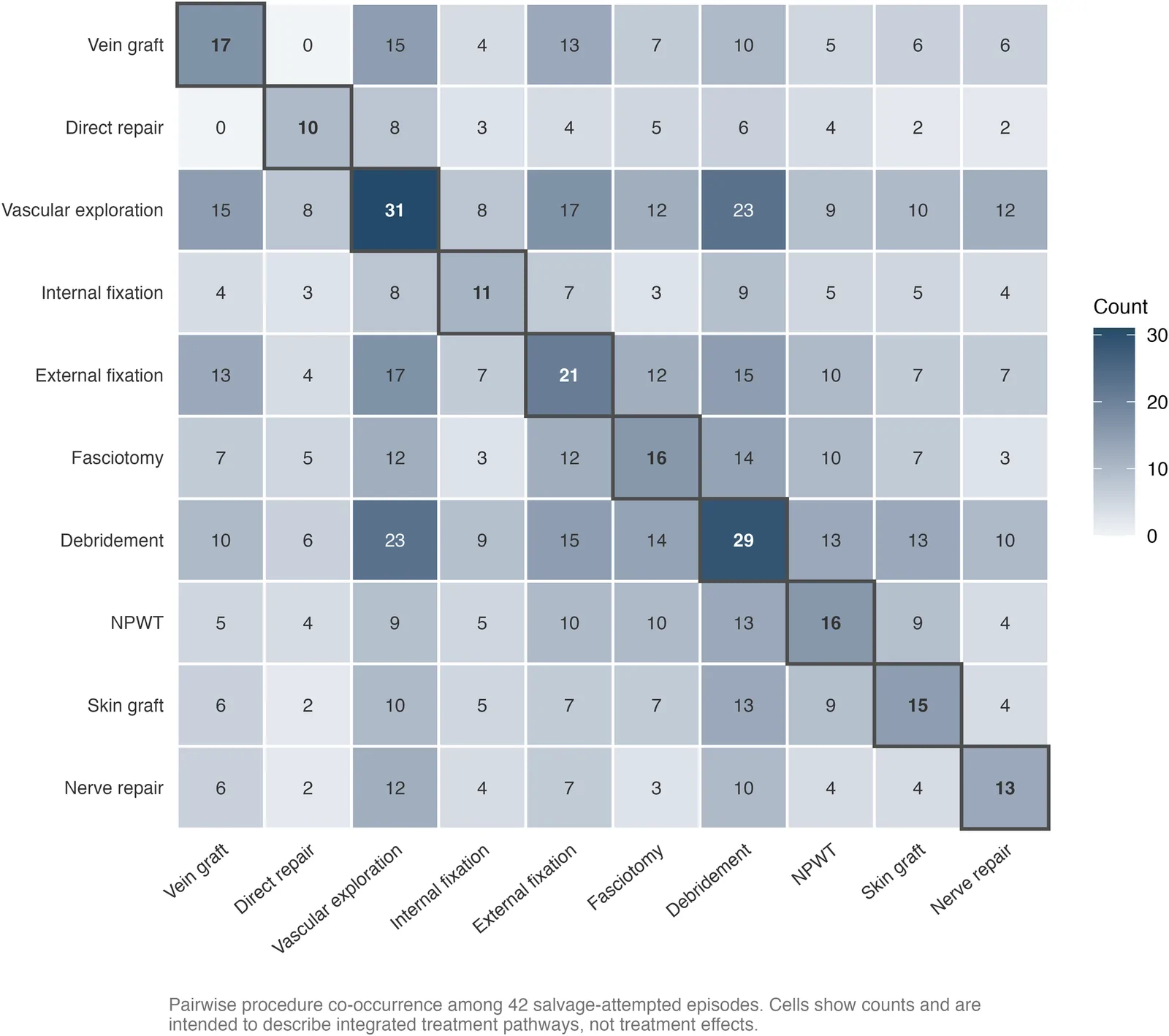
Procedure co-occurrence in salvage-attempted injury episodes. Heatmap showing pairwise co-occurrence of vascular, orthopedic, decompressive, and soft-tissue procedures among the 42 injury episodes in which limb salvage was attempted. Each cell represents the number of episodes in which both procedures were performed; diagonal cells indicate the overall frequency of each procedure. The matrix illustrates the integrated nature of multidisciplinary limb-salvage pathways and is intended as a descriptive representation of treatment patterns rather than treatment effects. NPWT, negative-pressure wound therapy.

These patterns illustrate the substantial overlap between vascular, orthopedic, decompressive, and soft-tissue procedures within individual salvage pathways.

## Limb outcomes after formal vascular reconstruction

Among the 27 salvage-attempted episodes treated with formal vascular reconstruction, 24 limbs were salvaged (88.9%) and three underwent secondary amputation (11.1%). Limb salvage was observed in 15 of 17 episodes treated with vein graft reconstruction (88.2%) and in 9 of 10 treated with direct arterial repair (90.0%) (Figure 5). The corresponding secondary-amputation rates were 11.8% and 10.0%, respectively.

**Figure 5:**
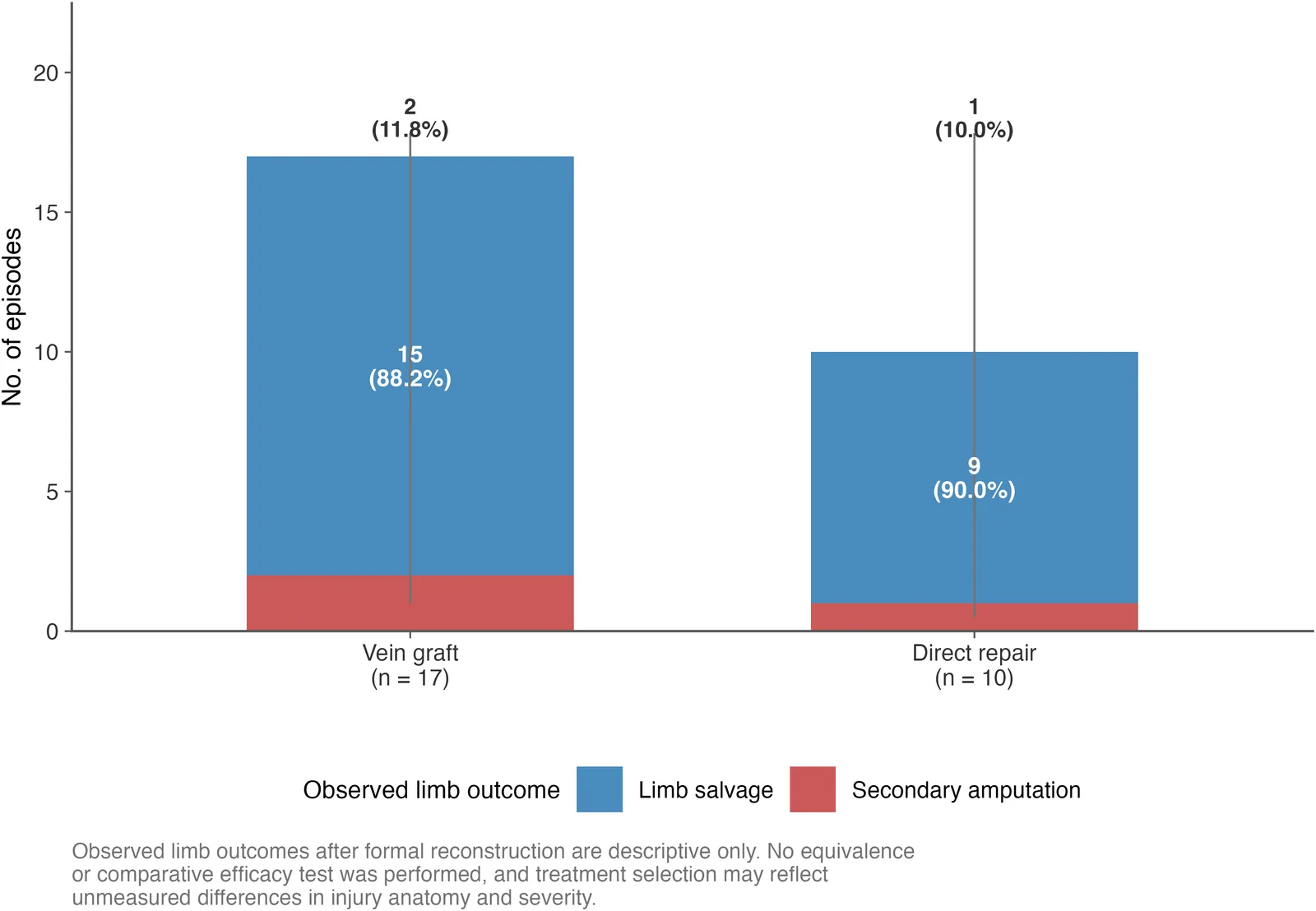
Observed limb outcomes after formal vascular reconstruction. Limb salvage and secondary amputation among salvage-attempted injury episodes treated with vein graft reconstruction (*n* = 17) or direct arterial repair (*n* = 10). Limb salvage was observed in 15 of 17 vein graft reconstructions (88.2%) and 9 of 10 direct repairs (90.0%). These outcomes are presented descriptively; no equivalence or comparative efficacy testing was performed between reconstruction techniques.

Because the reconstruction subgroups were small and treatment selection reflected clinical anatomy and injury characteristics that were not fully captured in the dataset, these rates are presented descriptively without formal comparison of efficacy between techniques.

## Admission laboratory findings

Availability of laboratory measurements within the prespecified 48-hour index-admission window was variable. PT was available for 33 of 46 episodes (71.7%), albumin for 23 (50.0%), and WBC, hemoglobin, platelet count, and NLR for 18 episodes each (39.1%). Accordingly, variable-specific missingness ranged from 28.3% to 60.9% (Supplementary Figure S1).

None of the evaluated laboratory parameters differed significantly between the limb-salvage and amputation groups. Median hemoglobin was 111 g/L (IQR, 93–127) among salvaged limbs and 88 g/L (IQR, 84–90) among amputations (*P* = 0.068). Median PT was 12.2 s (IQR, 11.4–13.0) and 13.5 s (IQR, 12.7–15.4), respectively (*P* = 0.074). The remaining laboratory comparisons were also nonsignificant (all *P* > 0.05). Given the extent of missing data, these findings were considered exploratory.

## Temporal patterns

Twenty-five injury episodes occurred during 2002–2012 and 21 during 2013–2025. The observed amputation rate was 16.0% in the early era and 23.8% in the recent era (*P* = 0.711), while primary amputation occurred in 8.0% and 9.5%, respectively (*P* = 1.000) (Figure 6A).

**Figure 6:**
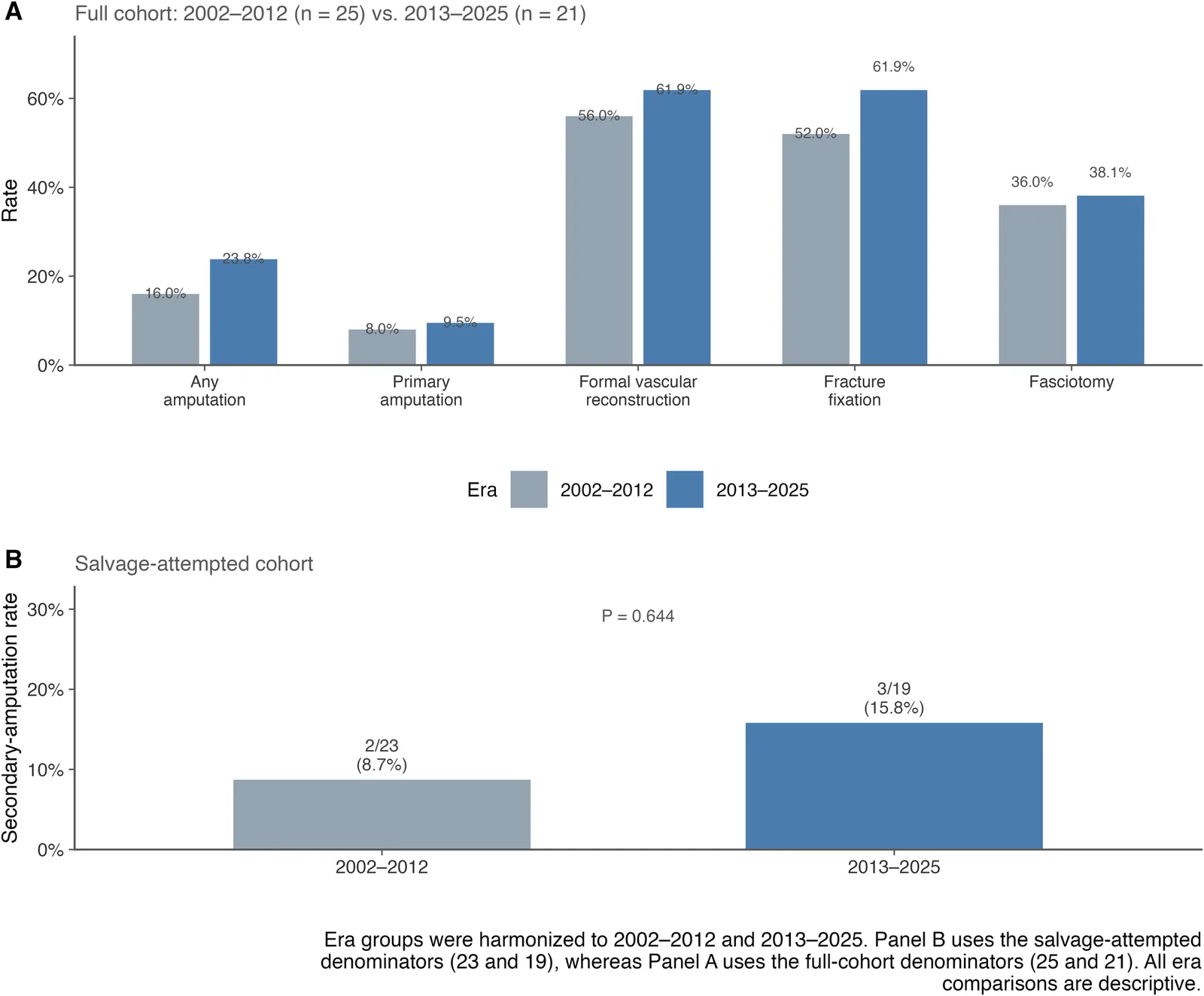
Treatment patterns and limb outcomes across eras. Comparison of treatment patterns and observed limb outcomes between 2002 and 2012 and 2013–2025. **(A)** Rates of any amputation, primary amputation, formal vascular reconstruction, fracture fixation, and fasciotomy in the full cohort (2002–2012, *n* = 25; 2013–2025, *n* = 21). **(B)** Secondary-amputation rates in the salvage-attempted cohort (2002–2012, *n* = 23; 2013–2025, *n* = 19). *P* values were calculated using Fisher's exact test. Temporal comparisons are descriptive and do not account for potential changes in case mix, referral patterns, or injury severity over the study period.

Formal vascular reconstruction was performed in 56.0% of early-era episodes and 61.9% of recent-era episodes (*P* = 0.769). The corresponding rates of fracture fixation were 52.0% and 61.9% (*P* = 0.561), and fasciotomy was performed in 36.0% and 38.1% (*P* = 1.000).

Within the salvage-attempted cohort, 23 episodes occurred in 2002–2012 and 19 in 2013–2025. Secondary amputation occurred in 2 of 23 early-era episodes (8.7%) and 3 of 19 recent-era episodes (15.8%; *P* = 0.644) (Figure 6B). No between-era comparison reached statistical significance.

## Discussion

In this retrospective single-center cohort of traumatic popliteal artery injury, reconstruction of the source records yielded 46 independent patient/injury episodes from 48 hospitalization records. Nine episodes ultimately resulted in amputation. Importantly, only four represented primary amputation without a documented preceding salvage attempt, whereas five occurred after limb salvage had been pursued. Among the 42 salvage-attempted episodes, 37 limbs were preserved, corresponding to an observed limb-salvage rate of 88.1%. These findings support a clinically useful distinction between primary amputation and failure of attempted salvage, rather than treating all amputations or subsequent hospitalizations as components of a single composite adverse outcome.

This distinction also changes how treatment patterns should be interpreted. Primary amputation reflects an early management pathway in which limb preservation was not pursued or was no longer considered feasible on the basis of the available record; it is not an appropriate comparator for reconstructive procedures intended to achieve salvage. Conversely, secondary amputation occurs after a salvage pathway has already been undertaken and is therefore the more relevant adverse outcome when examining associations with vascular reconstruction, fracture fixation, fasciotomy, and soft-tissue procedures. Restricting treatment-associated analyses to the salvage-attempted cohort reduces the structural bias introduced by including primary amputations, although it does not remove confounding related to injury severity, anatomy, or treatment selection.

## Vascular reconstruction and limb salvage

Formal vascular reconstruction was performed in 27 of the 42 salvage-attempted episodes. Limb salvage was observed in 15 of 17 episodes treated with vein graft reconstruction and in 9 of 10 treated with direct arterial repair. Thus, limb preservation was achieved in most patients undergoing either form of reconstruction. These observations are consistent with the broader vascular trauma literature emphasizing timely restoration of arterial inflow as a central component of limb salvage, together with skeletal stabilization and soft-tissue management (1, 4, 7, 8).

The present data, however, do not support a conclusion that vein graft and direct repair are equivalent or interchangeable in terms of efficacy. The two groups were small, and the choice of reconstruction technique was necessarily influenced by factors such as vessel defect length, local tissue condition, contamination, and the feasibility of a tension-free repair. Several of these determinants were not consistently available in the retrospective dataset. The observed salvage proportions should therefore be regarded as descriptive outcomes within two clinically selected treatment groups rather than as a direct comparison of techniques.

A related issue concerns episodes in which no formal reconstruction was documented. In the original analysis, vascular exploration alone, wound-management-only admissions, and absence of a recorded vascular procedure had been grouped together as “no reconstruction.” Review of the source data showed that these records represented different clinical circumstances, including isolated exploration, subsequent wound care, incomplete procedural documentation, and prior vascular treatment of uncertain type. They therefore cannot reasonably be interpreted as a single treatment strategy. In the revised analysis, these pathways are described separately and are not compared with vein graft or direct repair as though they represented alternative methods of revascularization. This distinction is particularly important because the available records do not consistently document the reason reconstruction was not performed or the status of distal perfusion at the time of treatment.

## Fasciotomy, fracture fixation, and treatment selection

Among the salvage-attempted episodes, secondary amputation occurred in 1 of 25 episodes with fracture fixation and in 4 of 17 without fixation. No secondary amputations occurred among the 16 episodes in which fasciotomy was performed, compared with 5 of 26 episodes without fasciotomy. A similar pattern was observed for skin grafting, whereas NPWT, debridement, and nerve repair showed little difference in observed secondary-amputation rates. None of these comparisons reached statistical significance.

These findings should not be interpreted as evidence that fracture fixation, fasciotomy, or skin grafting independently prevents amputation. Even after primary amputations are excluded, treatment allocation remains strongly influenced by clinical judgment. A limb considered suitable for continued reconstruction is more likely to undergo skeletal stabilization, decompression, repeated debridement, and definitive soft-tissue coverage than a limb in which salvage is failing. Conversely, fasciotomy may be preferentially performed in more severely ischemic limbs because of concern for reperfusion injury or compartment syndrome. The direction and magnitude of confounding by indication can therefore differ across procedures.

Fasciotomy nevertheless deserves specific clinical attention because compartment syndrome and reperfusion-related edema are established concerns after lower-extremity arterial injury (9–12). In this series, the absence of secondary amputation among fasciotomy-treated episodes is an observation worthy of further study, but the sample is too small to determine whether the difference reflects decompression itself, patient selection, timing of treatment, ischemia duration, or other aspects of the salvage pathway. The present findings therefore generate a hypothesis rather than support a treatment-effect estimate or a specific threshold for fasciotomy.

The same caution applies to fracture fixation. Skeletal stabilization is an integral component of the management of combined orthopedic and vascular trauma, both to restore limb alignment and to protect vascular reconstruction (13–18). However, the lower observed secondary-amputation rate among fixed fractures cannot be separated from the fact that fixation is itself part of a broader decision to continue limb reconstruction. Without standardized measures of fracture severity, ischemia time, soft-tissue destruction, and physiologic status, assigning an independent effect to fixation would be inappropriate.

## Integrated treatment pathways

One of the more informative findings of this study is the degree to which procedures occurred in combination. Vascular exploration frequently coincided with external fixation and debridement, while fasciotomy, NPWT, skin grafting, and nerve repair appeared within overlapping reconstructive pathways (Figure 4). For example, vascular exploration co-occurred with debridement in 23 salvage-attempted episodes and with external fixation in 17; fasciotomy and debridement occurred together in 14 episodes.

Limb salvage after popliteal artery injury is rarely attributable to a single operation, and the co-occurrence pattern reflects that reality rather than an analytic artifact. Revascularization must be coordinated with skeletal stabilization, compartment management, serial wound assessment, debridement, and soft-tissue coverage. The resulting procedural interdependence limits the usefulness of interpreting isolated procedures as independent predictors of outcome. The co-occurrence analysis therefore may be more informative as a description of how multidisciplinary care is actually delivered than as a preliminary screen for individual “protective” procedures (19, 20).

Larger datasets could examine these combinations more formally using pathway-based or cluster-based approaches rather than treating each operation as an isolated exposure. Such analyses would require substantially larger samples together with prospective recording of injury severity and treatment sequence.

## Admission laboratory findings

No admission laboratory variable differed significantly between the limb-salvage and amputation groups. Hemoglobin and PT showed the largest numerical differences, but neither reached conventional statistical significance. More importantly, laboratory availability was limited after application of the prespecified 48-hour index-admission window: missingness ranged from 28.3% for PT to 60.9% for several other variables.

Accordingly, A statistically null result here carries limited weight: these comparisons drew on small and variable subsets of the cohort, and the absence of significance is at least as likely to reflect that limitation as to indicate that early physiologic or hematologic disturbance has no bearing on limb outcome (19, 20). These analyses were based on small and variable subsets of the cohort and were retained primarily for descriptive purposes. Future studies would benefit from systematic collection of early hemoglobin, coagulation, inflammatory, and metabolic parameters using standardized sampling windows.

## Temporal patterns

The 23-year study period spans substantial changes in vascular imaging, reconstructive techniques, orthopedic fixation, wound management, and thresholds for attempted limb salvage. In the present cohort, formal vascular reconstruction, fracture fixation, and fasciotomy were numerically more frequent in 2013–2025 than in 2002–2012, while the observed amputation rate also increased from 16.0% to 23.8%. Within the salvage-attempted cohort, secondary amputation occurred in 8.7% of early-era episodes and 15.8% of recent-era episodes. None of these between-era differences was statistically significant.

These temporal comparisons should remain descriptive. The dataset does not contain sufficiently complete information on changes in referral patterns, injury complexity, ischemia duration, or institutional treatment thresholds to explain the observed differences. The era analysis therefore demonstrates variation in practice over time but cannot determine whether these changes reflect differences in case mix, treatment philosophy, or quality of care.

## Clinical interpretation

The revised analysis supports three practical observations. Primary amputation and secondary amputation after attempted salvage represent different clinical situations that should be reported separately; among patients in whom salvage was attempted, limb preservation was achieved in 37 of 42 episodes, indicating that successful salvage was common despite the complexity of popliteal artery injury.

Formal vascular reconstruction, moreover, formed only one component of treatment. The high degree of procedural co-occurrence indicates that management is better understood as a coordinated sequence of vascular, orthopedic, decompressive, and soft-tissue interventions than as a collection of independent procedures—a distinction that matters when interpreting retrospective associations between individual operations and outcome.

The lower observed secondary-amputation rates among patients undergoing fasciotomy or fracture fixation should be read as signals for future investigation rather than evidence of treatment benefit. Confirming any independent effect would require prospective data on ischemia duration, hemodynamic status, fracture severity, venous and nerve injury, compartment pressures, soft-tissue loss, contamination, vascular lesion characteristics, and the timing and sequence of individual procedures.

## Limitations

This study has several limitations. The most consequential is sample size: the final analysis included 46 independent injury episodes, with nine amputations overall and only five secondary amputations among the 42 salvage-attempted episodes. These event counts precluded reliable multivariable modeling and limited the precision of all procedure-specific comparisons; the results should therefore be regarded as descriptive and hypothesis-generating.

The retrospective design, spanning 23 years, is a related concern. Although reconstructing hospitalization records into patient/injury episodes reduced the dependency created by repeated admissions from the same injury, the completeness of historical documentation varied, vascular procedure classification required review of free-text operative notes, and some pathways remained incompletely specified—in particular, the reasons for not performing formal reconstruction and objective measures of distal perfusion.

Several major determinants of limb viability were also not systematically captured, including ischemia duration, detailed vascular lesion morphology, concomitant venous injury, severity of nerve injury, fracture classification, extent of soft-tissue loss, contamination, compartment pressures, and hemodynamic status. Although all injuries involved the popliteal artery, more granular anatomic information on the precise location and extent of arterial damage was not consistently recorded, so residual confounding by injury severity and indication is unavoidable.

Laboratory data compounded this incompleteness: despite use of a strict 48-hour sampling window, missingness ranged from 28.3% to 60.9%, and multiple imputation was not undertaken given the small cohort and the likely non-random nature of the missingness. Laboratory comparisons therefore carry limited precision.

Outcome ascertainment relied primarily on the institutional record. One injury-related readmission requiring further intervention was identified, but care received at outside institutions could not be systematically captured, and the study lacked uniform long-term assessment of vascular patency, functional recovery, chronic pain, return to work, or patient-reported outcomes—limb salvage should not be equated with good long-term limb function.

Individual procedures were, finally, highly interdependent. The co-occurrence patterns in Figure 4 illustrate why unadjusted comparisons of fasciotomy, fracture fixation, or soft-tissue procedures cannot establish independent causal effects—a limitation intrinsic to the dataset, and a principal reason the revised analysis emphasizes observed treatment pathways over procedural efficacy.

## Strength and scope of the study

Despite these limitations, the study provides a longitudinal description of multidisciplinary management in a clinically homogeneous cohort of traumatic popliteal artery injuries. Reconstructing repeated hospitalization records into independent injury episodes allowed primary amputation, failed salvage, and readmission to be distinguished more clearly than in the original event-level analysis. The study also captures vascular, orthopedic, decompressive, and soft-tissue procedures within the same analytic framework, which helps illustrate how limb salvage is delivered in routine multidisciplinary practice.

The findings are most appropriately viewed as a detailed single-center description of treatment pathways and observed limb outcomes rather than as a comparative effectiveness study. Their main value lies in showing that primary amputation, secondary amputation, and multidisciplinary treatment pathways are analytically distinguishable categories worth separating in future popliteal-injury cohorts—particularly ones large enough to adjust for ischemia time and injury severity.

## Conclusion

In this single-center retrospective cohort of traumatic popliteal artery injury, primary and secondary amputations represented distinct clinical pathways. Among the 42 injury episodes in which limb salvage was attempted, 37 limbs were preserved, corresponding to an observed salvage rate of 88.1%. High limb-salvage proportions were observed after both vein graft reconstruction and direct arterial repair, but the small, clinically selected treatment groups do not permit conclusions regarding comparative efficacy. The marked co-occurrence of vascular, orthopedic, decompressive, and soft-tissue procedures underscores the integrated nature of limb-salvage care. Procedure-specific differences in observed secondary-amputation rates should be considered exploratory and require confirmation in larger cohorts with standardized measures of injury severity, ischemia duration, and treatment timing.

## Data Availability

The original contributions presented in the study are included in the article/Supplementary Material, further inquiries can be directed to the corresponding author.
